# Christmas Entertainments at Hospitals

**Published:** 1887-12-31

**Authors:** 


### CHRISTMAS ENTERTAINMENTS AT HOSPITALS.

We have received several interesting accounts of Christmas festivities which have taken place at the various hospitals, and for these we beg to thank our correspondents. Unfortunately, in every case they have reached us too late for publication in this week's issue. Next week we shall devote as much space to this subject as the pressure of other matters will permit. The entertainments in many cases have been of a highly pleasing character.

